# Molecular evolution and diversity of isomerase–reductase clusters involved in the bacterial metabolism of glycosaminoglycans

**DOI:** 10.1128/msphere.00817-25

**Published:** 2025-12-29

**Authors:** Yu Nishimura, Kenji Okumura, Sayoko Oiki, Kohei Ogura, Wataru Hashimoto

**Affiliations:** 1 Laboratory of Basic and Applied Molecular Biotechnology, Division of Food Science and Biotechnology, Graduate School of Agriculture, Kyoto University 222734, Uji, Kyoto, Japan; The University of Iowa, Iowa City, Iowa, USA

**Keywords:** 4-deoxy-l-*threo*-5-hexosulose uronate, glycosaminoglycan, hidden Markov model, nonhomologous isofunctional enzymes, cluster, phylogenetic analysis

## Abstract

Glycosaminoglycans (GAGs), comprising uronic acids and amino sugars, are widely distributed in human tissues such as the intestine and oral cavity. Various bacteria colonize these tissues by assimilating GAGs. During GAG degradation, 4-deoxy-l-*threo*-5-hexosulose uronate (DHU) is produced. Pectin, an abundant plant component, is also degraded into DHU. DHU is metabolized in a stepwise manner by the isomerase KduI or its nonhomologous isofunctional enzyme DhuI, followed by the reductase KduD or DhuD, belonging to the same reductase–dehydrogenase family. Previous studies have found that the genes encoding isomerase and reductase (*kduI–kduD* and *dhuD–dhuI*, respectively) are usually organized in clusters. Therefore, it was believed that the *kduI–kduD* and *dhuD–dhuI* clusters evolved independently. However, the discovery of a hybrid *kduI–dhuD* cluster raised questions regarding the evolution of these clusters. This study investigated the diversity of clusters through a pan-genomic phylogenetic analysis across 3,550 bacterial strains. Among 16 possible cluster structures, 10 types were involved in DHU metabolism. Bacteroidota possessed a hybrid-type *kduI–dhuD* cluster, while Bacillota, but not Pseudomonadota or Bacteroidota, possessed the cluster *dhuD–dhuI*. Using public data sets from the human fecal microbiome and environmental habitats, we detected the prevalence of *kduI–dhuD* and *dhuD–dhuI* clusters in gut microbes. Although DHU is generated from oligomerized GAG degradation by unsaturated glucuronyl hydrolase (UGL), the UGL gene was frequently found in pathogenic strains containing *kduD–kduI*, *dhuD–dhuI*, *kduI–dhuD*, or *dhuD–kduI*, indicating that the acquisition of these clusters is advantageous for human colonization.

**IMPORTANCE:**

Glycosaminoglycans (GAGs), crucial components of the extracellular matrix, play vital roles in host infection by pathogenic bacteria and host colonization by commensal bacteria. The *dhuD–dhuI* cluster is well conserved within certain phyla, and it appears to have a strong association with GAG metabolism. In contrast, *kduI*-containing clusters are more widely distributed across bacterial species. Based on the possession ratios of genes encoding the enzymes involved in the production of 4-deoxy-l-*threo*-5-hexosulose uronate, this study indicates that the substrates differ depending on the specific cluster type.

## INTRODUCTION

Glycosaminoglycans (GAGs) are linear polysaccharides composed of uronic acids and amino sugars that play vital roles in the extracellular matrices of animals (1). Major types of GAGs include nonsulfated hyaluronic acid (composed of glucuronic acid and *N*-acetylglucosamine), chondroitin sulfate and dermatan sulfate (glucuronic acid or iduronic acid and *N*-acetylgalactosamine with sulfation at various positions), heparin/heparan sulfate (repeating units of uronic acid and glucosamine with varying degrees of sulfation), and keratan sulfate (unique among GAGs because it contains galactose instead of uronic acid, along with *N*-acetylglucosamine) (2).

**Fig 1:**
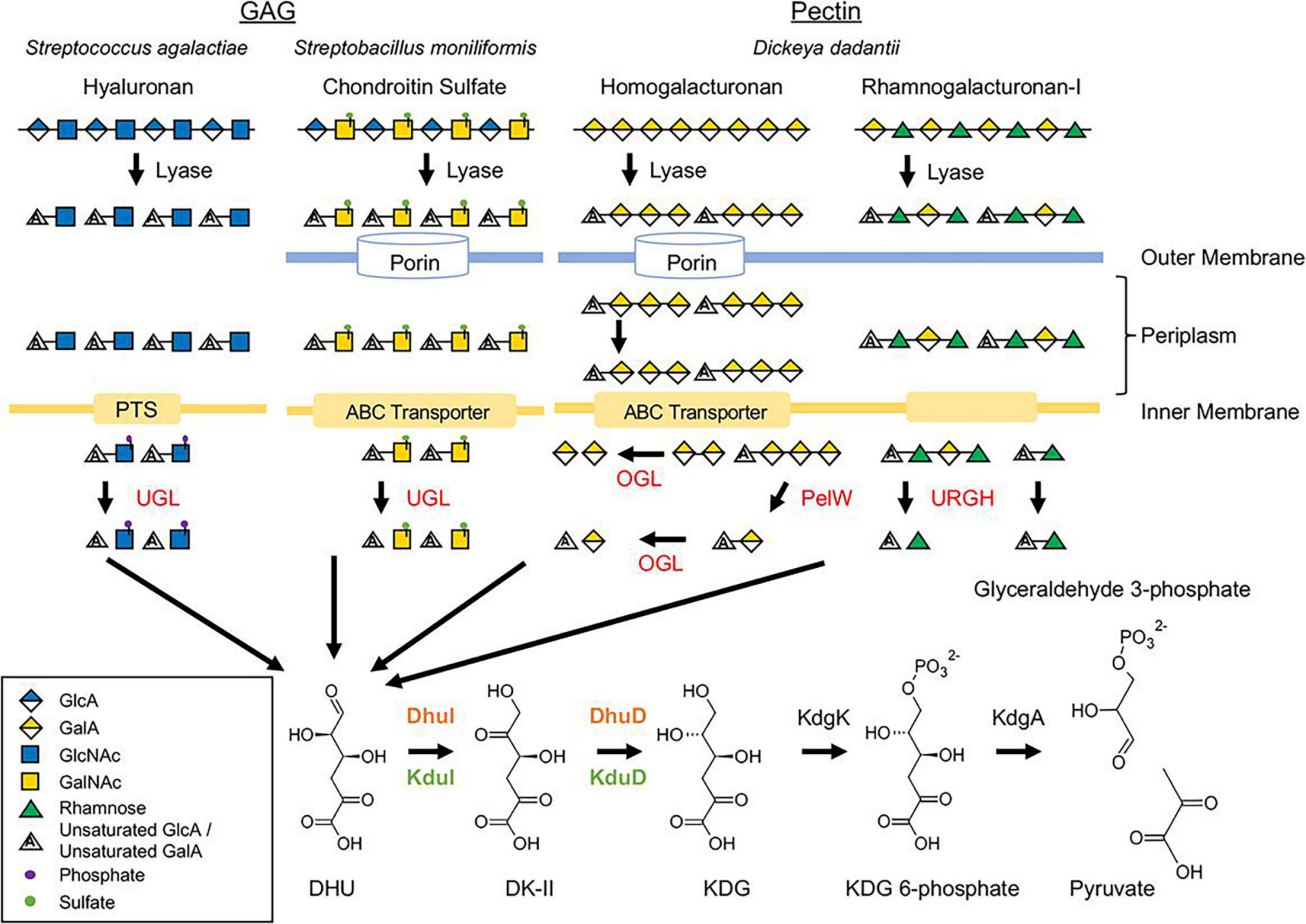
Metabolism of GAGs and pectins. Metabolism of GAGs and pectins. The names of saccharides were described according to a previous report (12).

Specific pathogenic bacteria, such as *Streptococcus pneumoniae* and *Streptococcus dysgalactiae*, have evolved specialized gene clusters for degrading and utilizing GAGs, particularly hyaluronic acid (3–5). This process is crucial for bacterial pathogenicity because it involves the extracellular degradation of hyaluronic acid by hyaluronate lyase, followed by intracellular transport and degradation of unsaturated disaccharides. GAGs are also important for mediating interactions between the host and gut bacteria (6, 7). The unsaturated disaccharides produced by the lyase reaction are transported into the cytoplasm via the phosphotransferase system or ATP-binding cassette transporters (4, 8, 9) (Fig. 1). In *Streptococcus* species, disaccharides transported by the phosphotransferase system are degraded by unsaturated glucuronyl hydrolases (UGLs) belonging to glycoside hydrolase family 88 (8, 10). These UGLs degrade unsaturated disaccharides into unsaturated glucuronic acid and amino sugars. The unsaturated glucuronic acid undergoes nonenzymatic ring opening, producing 4-deoxy-l-*threo*-5-hexosulose uronate (DHU). In some bacteria, the enzyme KdgF facilitates this ring opening reaction enzymatically (11).

Pectin, a heteropolysaccharide abundant in the plant cell walls, is primarily composed of α-1,4-d-galacturonic acid units and contains various neutral sugars such as rhamnose, arabinose, galactose, and xylose (13). Pectin lyases are produced by various bacteria such as the plant pathogen *Dickeya dadantii*, the human gut bacterium *Bacteroides thetaiotaomicron*, and the human pathogen *Yersinia enterocolitica* (14). In *D. dadantii*, pectin oligosaccharides produced by pectin lyase are transported into the periplasm via porins KdgM and/or KdgN (15–17), followed by passage into the cytoplasm through the ATP-binding cassette transporter TogMNAB (18, 19). In the cytoplasm, oligogalacturonate lyases (OGLs) catalyze the conversion of saturated and unsaturated digalacturonates into monogalacturonate and DHU (20) (Fig. 1). Thus, DHU is produced from GAGs and pectin. In *D. dadantii*, DHU is also generated from unsaturated rhamnogalacturonan by unsaturated galacturonyl hydrolase (YteR), a member of the GH105 family (21).

**Fig 2:**
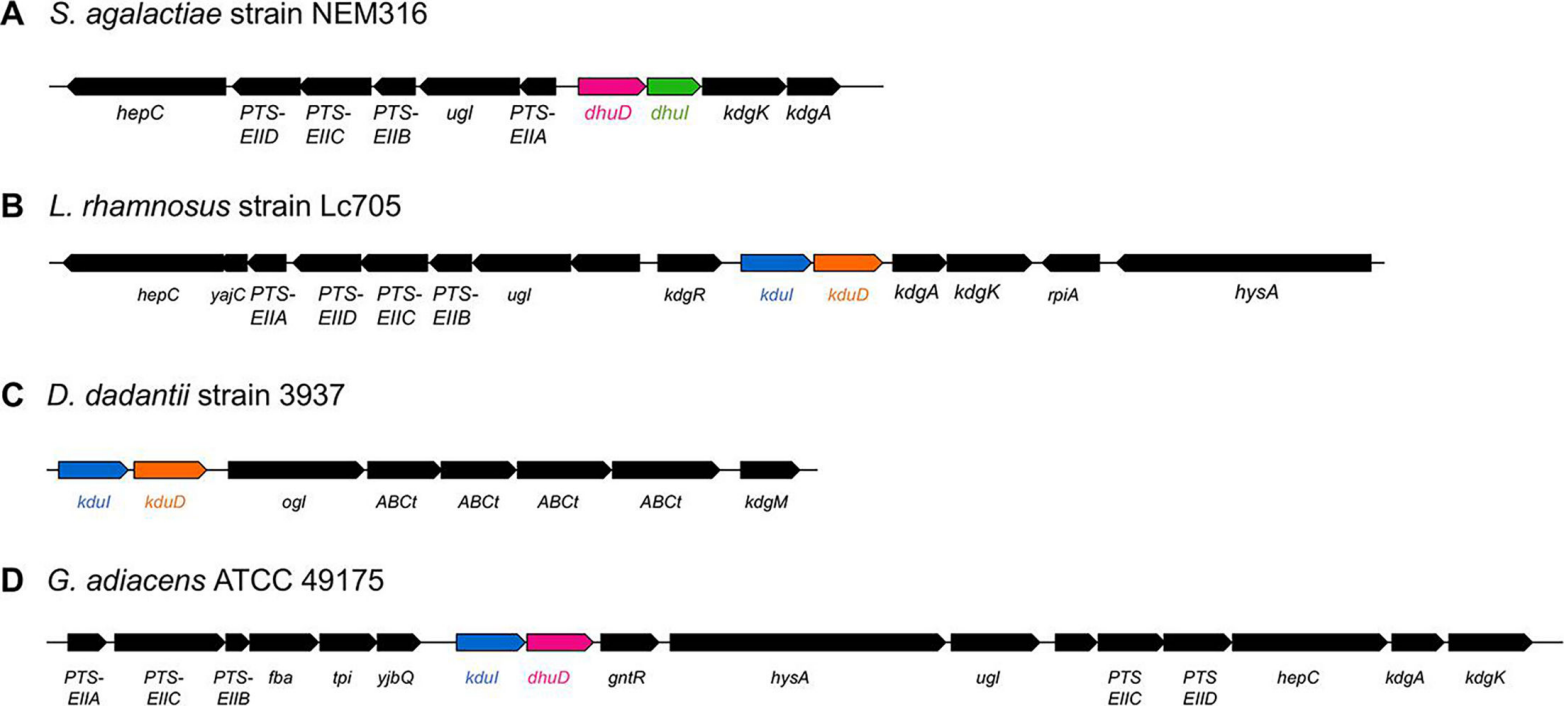
Gene clusters involved in the metabolism of GAGs and pectins. GAG-metabolizing gene clusters of (**A**) *S. agalactiae* strain NEM316 (NC_004368.1), (**B**) *L. rhamnosus* strain Lc705 (NC_013199.1), (**C**) *D*. *dadantii* strain 3937 (NC_014500.1), and (**D**) *G. adiacens* ATCC 49175 (GG694015.1). The term “*ABCt*” refers to genes that encode components of an ABC transporter. The illustrations were created using the drawGeneArrows3 tool, developed by Dr. Yoshiyuki Ohtsubo from Tohoku University (https://www.ige.tohoku.ac.jp/joho/).

In *Streptococcus agalactiae*, DHU is isomerized by DhuI into 3-deoxy-d-glycero-2,5-hexodiulosonate (DK-II), which is further reduced by the NADH-dependent reductase DhuD to form 2-keto-3-deoxy-d-gluconate (22). The enzymes DhuD and DhuI are encoded by the cluster *dhuD–dhuI* in the *S. agalactiae* genome (Fig. 2A). The probiotic *Lacticaseibacillus rhamnosus* and the plant pathogen *D. dadantii* produce the isomerase KduI and the NADH-dependent reductase KduD, whose genes also exist in a cluster as *kduI–kduD* (23) (Fig. 2B and C). The genes *dhuI* and *kduI* encode enzymes that catalyze similar reactions but lack sequence homology, indicating the presence of nonhomologous isofunctional enzymes. KduI and DhuI belong to the KduI/IolB isomerase family (InterPro ID, IPR021120) and the RpiB/LacA/LacB sugar-phosphate isomerase family (IPR003500), respectively. Although KduD and DhuD belong to the short-chain dehydrogenase–reductase family (IPR002347), their sequence identity is low. For example, KduD from *L. rhamnosus* only exhibit 42.2% identity with DhuD from *S. agalactiae*. Therefore, the clusters *dhuD–dhuI* and *kduI–kduD* are believed to have evolved independently. However, a hybrid cluster *kduI–dhuD* was recently detected in *Granulicatella adiacens*, isolated from the oral cavity (Fig. 2D) (24). This discovery of a hybrid cluster raised questions about the evolutionary process of cluster formation.

The ability of microbes to metabolize diverse saccharides provides a significant evolutionary advantage, enabling them to thrive in environments in which preferred carbon sources are scarce. This pressure has led to the development of highly specialized operons for different sugars. Kaznadzey et al. (25) reported that approximately 50% of studied genes are co-localized in bacterial genomes with other carbohydrate metabolism genes and that the preference toward the co-localization of carbohydrate metabolism genes varies between 40% and 76% for bacterial taxa. Bacteria can acquire entire operons or gene clusters involved in saccharide metabolism from other bacteria through horizontal gene transfer (26). Duplication of genes followed by divergence of the duplicated copies can lead to novel genes with altered substrate specificities or catalytic activities within a saccharide metabolic pathway, which are then co-opted into existing or novel operons or gene clusters (27).

Although nonhomologous isofunctional enzymes are well documented, only a few studies have investigated the formation of gene clusters encoding nonhomologous isofunctional enzymes catalyzing the same sequential reaction. Furthermore, there has been limited research on the diversity of cluster structures across bacterial genomes. Our investigation focused on cluster formation and structure, particularly examining clusters related to the metabolism of GAGs. Hence, this study aimed to classify cluster structures across bacterial genomes and highlight the metabolism of GAGs and the intriguing formation of clusters encoding nonhomologous isofunctional enzymes. Understanding these complex genetic arrangements provides valuable insights into the evolutionary pressures shaping bacterial metabolism and cluster architecture.

## RESULTS AND DISCUSSION

### Variations in isomerase and reductase clusters across bacterial genomes

**Fig 3:**
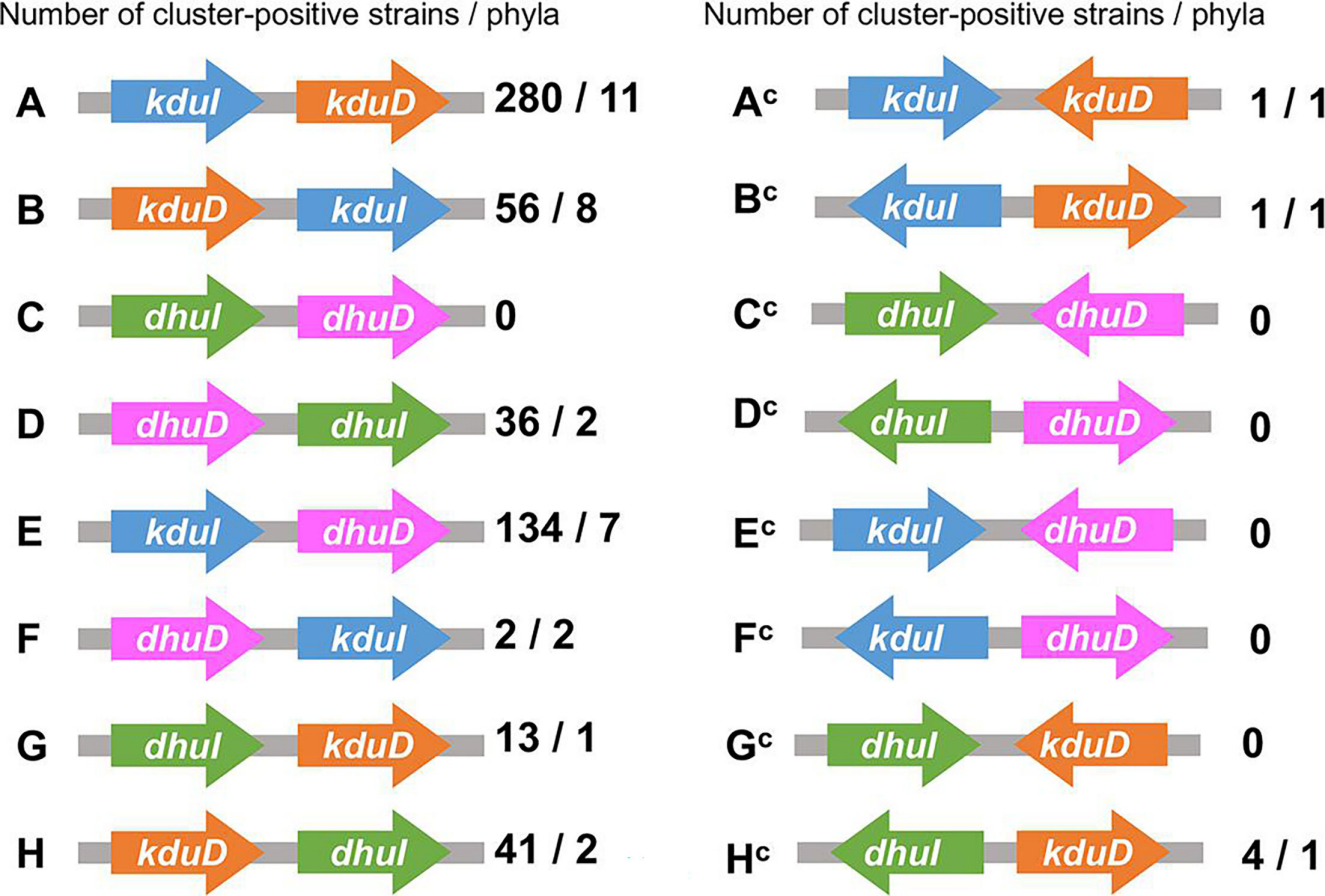
Possible configurations of isomerase–reductase clusters. Types A–H contain genes encoding isomerases and reductases oriented in the same direction. Types A^C^–H^C^ represent clusters in which the two genes are arranged in opposite directions. The number of cluster-positive strains and phyla is presented to the right of each cluster diagram.

**Fig 4:**
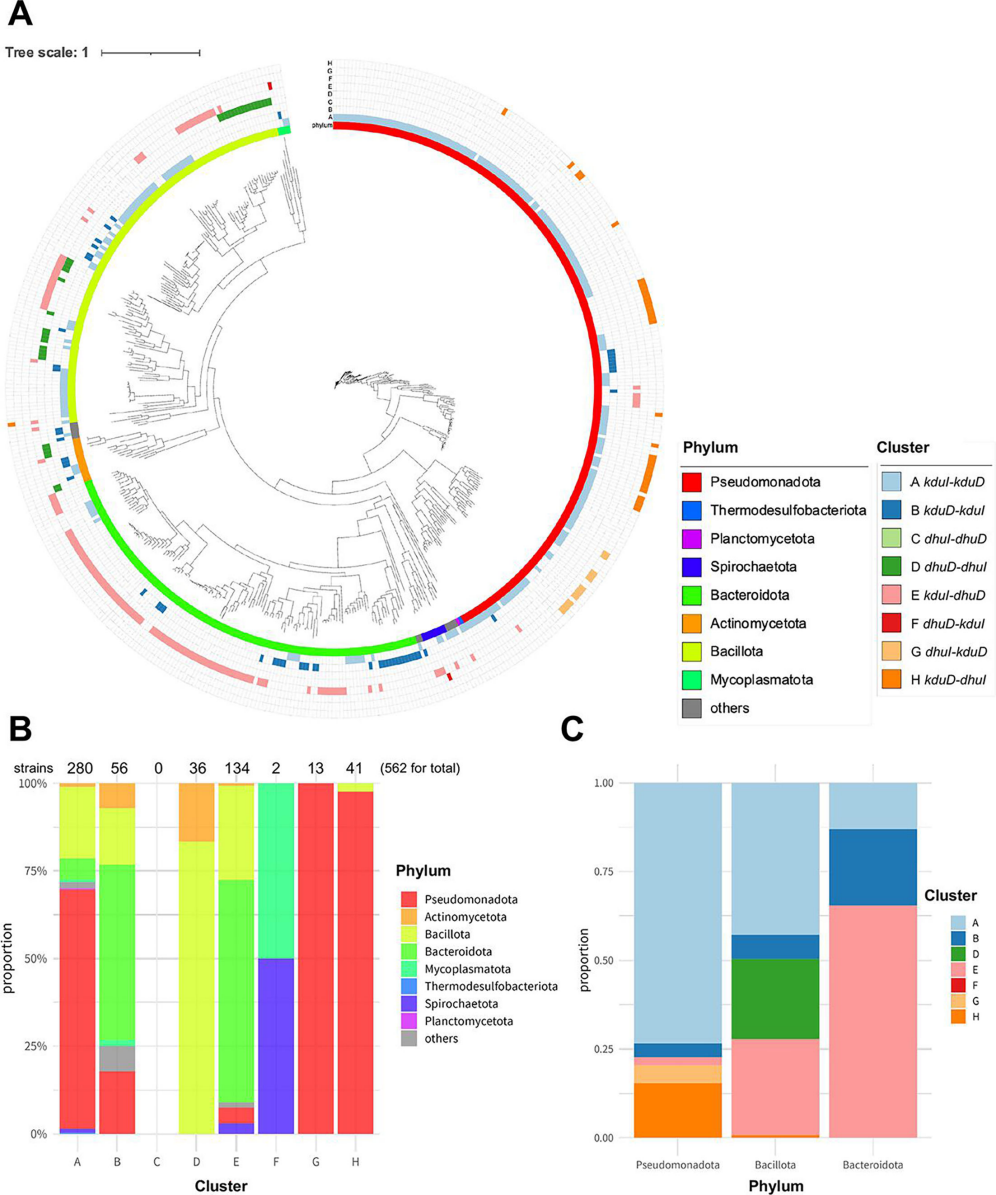
Pan-genome analysis of the cluster types. (**A**) A phylogenetic tree based on core genomes and cluster types (types A–H). The figure was drawn using iTOL (version 6) (30). (**B**) Proportions of phyla in each type. The numbers above the bar graphs represent the total number of strains harboring each cluster. (**C**) Proportions of cluster types in the phyla Pseudomonadota, Bacteroidota, and Bacillota.

There are 16 potential combinations (types A–H and A^c^–H^c^) of clusters encoding isomerases (KduI or DhuI) and reductases (KduD or DhuD), as illustrated in Fig. 3 and 4. Using representative sequences from 3,550 bacterial strains (Table S1), DHU isomerases and DK-II reductases were identified by BLAST and hidden Markov model (HMM) searches (28, 29). Among the 3,550 representative genomes, 568 clusters were identified, and 538 strains (15%) harbored putative clusters, indicating that some strains possess two or more clusters in their genomes. Among the 16 possible types of cluster combinations, 10 were identified in bacterial genomes (Fig. 3 and 4). As most of the clusters (562 of 568 clusters) arranged the two genes with the same direction (types A–H), we focused on all cluster types excluding C in the subsequent analysis. Among the seven investigated types, strains harboring the cluster *kduI–kduD* (type A) were most common (280 strains and 11 phyla; Tables S3 and S4), followed by those harboring *kduI–dhuD* (type E; 134 strains and seven phyla; Fig. 4B; Tables S3 and S7). The cluster *kduD–kduI* (type B), in which the order of the two genes is reversed from that in type A clusters, was widely distributed independent of the phylum and type A clusters (Tables S3 and S5). The cluster *dhuD–dhuI* (type D) was detected only in the phyla Bacillota (30 strains) and Actinomycetota (six strains; Tables S3 and S6). Moreover, 90% of strains possessing the hybrid cluster *kduI–dhuD* (type E) belonged to either Bacteroidota (63%) or Bacillota (27%; Fig. 4C; Table S3 and S7). The type F cluster *dhuD–kduI* was detected specifically in the phyla Mycoplasmatota and Spirochaetota (Fig. 4B; Tables S3 and S8). Strains harboring the hybrid *dhuI–kduD* cluster (type G) were limited to the families Sphingomonadaceae (nine strains) *and* Erythrobacteraceae (four strains) in the phylum Pseudomonadota (Fig. 4C; Tables S3 and S9). Type H clusters were primarily detected in the phylum Pseudomonadota, but they were also present in *Faecalibacterium duncaniae* (strain JCM 31915, phylum Bacillota). The cluster *kduD–dhuI* (type H) was detected in 41 strains, of which 40 belonged to the phylum Pseudomonadota (Tables S3 and S10). Approximately 18% of strains (243 of 1,378 strains) belonging to Pseudomonadota harbored clusters of any type, primarily types A (13.9%) and H (2.9%).

Regarding DHU production, Hobbs et al. (11) reported that acquisition of *kdgF* provides an advantage by enabling enzymatic ring opening of unsaturated glucuronic acid. Among the 538 cluster-positive strains, the proportion of *kdgF*-positive strains was <50% for all types excluding type E-positive strains (Fig. S1A), indicating that in most strains, unsaturated glucuronic acids are converted to DHU nonenzymatically in the absence of KdgF.

Among type A–H clusters (562 detected clusters) , 552 (98%) and 552 (98%) clusters co-occurred with *kdgA* and *kdgK*, respectively, which encode the DHU-metabolizing enzymes 2-dehydro-3-deoxyphosphogluconate aldolase and 2-dehydro-3-deoxygluconokinase, respectively (Fig. S1B and Table S14), suggesting that the cluster-possessing strains metabolizes DHU after enzymatic reaction by isomerases and reductases.

Of the 3,550 strains analyzed, 3,012 lacked DHU metabolism–related gene clusters. Among these, *kduI* was detected in 201 strains, all of which carried *kduD. dhuI* was identified in 144 strains, of which 124 and 141 strains also harbored *dhuD* and *kduD*, respectively. This suggests that these strains retain isomerase and reductase genes at separate loci on the chromosome rather than as part of an operon or gene cluster. Additionally, 73 strains, including those from the genus *Mycoplasma*, lacked *kduI*, *kduD*, *dhuI*, and *dhuD*.

**Fig 5:**
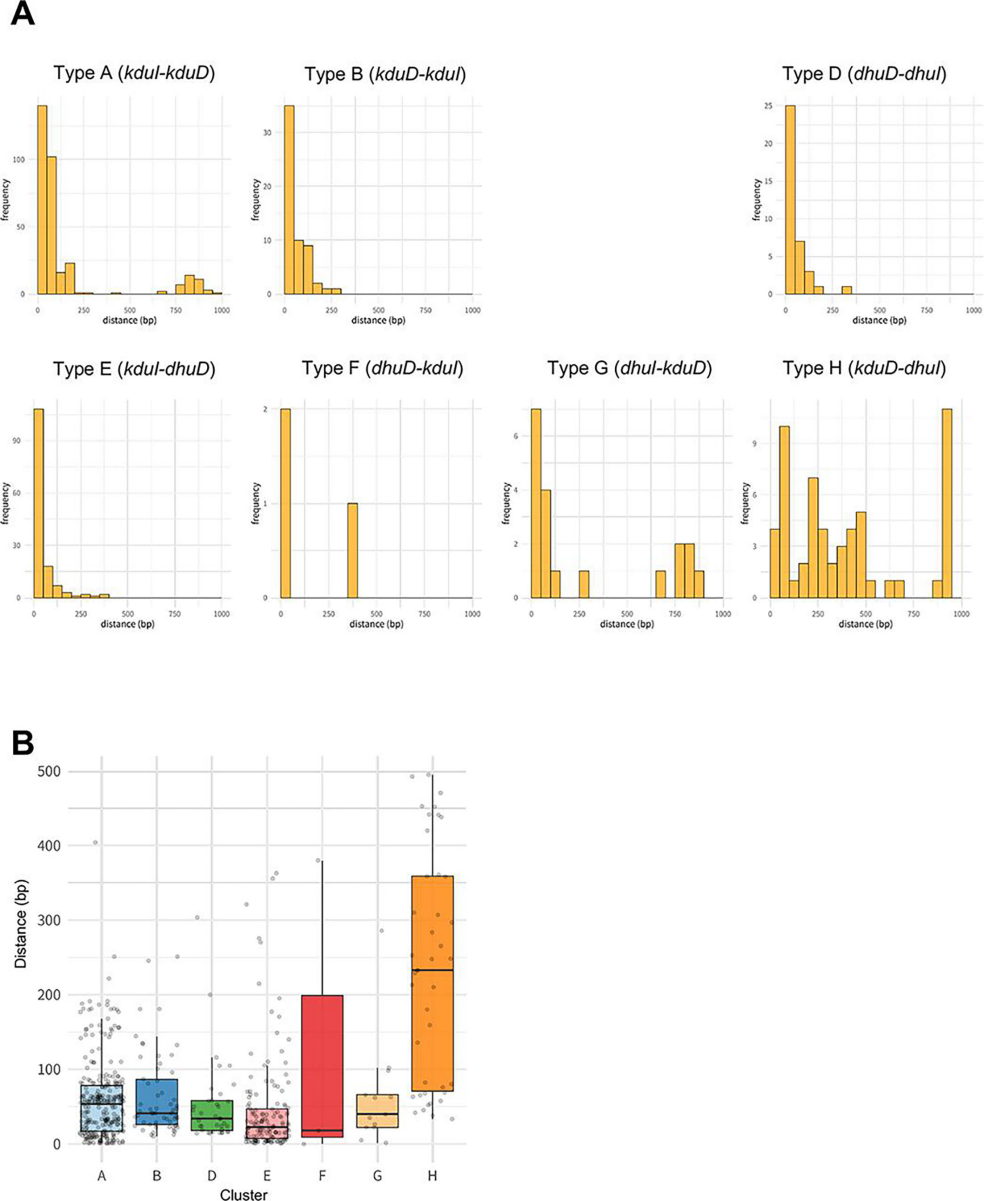
Distance between isomerase and reductase genes. (**A**) Bar plot of distance between genes and frequency with a cutoff of 1000 bp for each cluster (types A–H). (**B**) Box plots of distance for each cluster with a cutoff of 500 bp.

To explore whether isomerase and reductase genes are organized as clusters, we calculated the genomic distance between the two genes (Fig. 5), which revealed a close distance for all types, excluding type H (*kduD–dhuI* cluster). Of the 41 type H strains, 12 carried a gene encoding a protein with a cupin-domain protein located between *kduD* and *dhuI*. These proteins, characterized by the presence of a cupin domain, were identified as KdgF, which facilitates the transformation of cyclic unsaturated uronates into their linear ketone forms (29), opposed to KduI, which exhibits similarity to cupin-type phosphoglucose isomerase (31). Furthermore, in 13 of the 41 strains, a gene identified as *kduI* was located upstream of *kduD*, forming a tandem cluster *kduI–kduD–dhuI*, whereas the remaining 28 strains harbored simple type H clusters in the absence of *kduI*. These data suggested that type H clusters were formed through different evolutionary processes.

### Insights into the phyla Bacteroidota and Bacillota

We further analyzed the diversity of clusters in the phyla Bacteroidota and Bacillota, in which strains bearing the type E hybrid cluster were abundant (Fig. 4C; Table S1). Bacteroidota contained type A, B, or H clusters, whereas one of the strains (*Wenyingzhuangia fucanilytica* strain CZ1127) harbored a tandem cluster composed of *kduD–kduI–dhuD*. Compared with that in other phyla, the possession ratio of type A clusters was low in Bacteroidota (17 of 309 strains). Bacillota contains the families Streptococcaceae (15 of 51 strains were cluster-positive), Clostridiaceae (14 of 41 strains were cluster-positive), and Lachnospiraceae (17 of 34 strains were cluster-positive), which are common residents in the oral cavities and guts of humans and animals. By contrast, no cluster-positive strains were detected within Staphylococcaceae, a family typically residing in the nasal cavity. Although *Staphylococcus aureus* secretes hyaluronate lyases, these lyases are involved in the dispersion of hyaluronic acid–containing biofilms and subsequent distribution of the pathogenic bacterium to host tissues, rather than the utilization of hyaluronic acid as a nutrient source (32). Streptococcaceae and Clostridiaceae harbored primarily type D (dhuD–dhuI) clusters. Five strains in the family Lachnospiraceae (*Marvinbryantia formatexigens* and four *Blautia* species) harbored the tandem *kduI–dhuD–dhuI* cluster.

### Correlation with DHU-producing enzymes

Based on the observed habitat preferences, we hypothesized that the possession of GAG- and pectin-degrading enzymes could explain the patterns of environmental adaptation. Oligomerized GAGs are degraded by UGLs, whereas pectins are cleaved by OGLs and/or YteRs. Enzymatic reactions catalyzed by UGLs, OGLs, and YteRs commonly result in the production of DHU, which serves as the substrate of the isomerases KduI and DhuI. Therefore, the presence of UGLs, OGLs, and YteRs is suspected to be associated with the environmental adaptation of bacteria. We compared the rates of UGL, OGL, and YteR gene positivity among the seven types of clusters (Fig. S1C). Strains containing type B, D, E, or F clusters harbored UGL genes at high rates, indicating that the acquisition of these clusters is advantageous for colonization of human hosts. Although OGLs and YteRs target oligomerized pectin, the presence of clusters in the genome varied among type D-, E-, and F-positive strains.

*M. formatexigens* (DSM 14469), a human gut-commensal anaerobe bacterium, harbors *kduI–dhuD–dhuI*, which is positive for types D (*dhuD–dhuI*) and E (*kduI–dhuD*), as well as a UGL gene, whereas it lacks OGL and YteR genes. Although the UGL gene was detected in type D-positive strains, none of them harbored the OGL gene. However, a previous study demonstrated that this bacterium harbors 103 GHs, including GH28 family hydrolases (polygalacturonases), which are involved in pectin digestion (33), indicating that pectin is degraded into saturated galacturonic acid and metabolized independen tly of KduI or DhuI. In addition to *M. formatexigens*, the *kduI–dhuD–dhuI* cluster was detected in four *Blautia* species, namely, *Blautia producta* (DSM 2950), *Blautia pseudococcoides* (strain YL58), *Blautia hansenii* (DSM 20583), and *Blautia argi* (KCTC 15426). UGL genes were detected in three *Blautia* species (*B. producta*, *B. pseudococcoides*, and *B. hansenii*), and *B. producta* also harbored YteR, suggesting that these three species can metabolize GAGs. None of the *Blautia* species harbored an OGL gene. It remains unclear whether *Blautia* species utilize pectin as a nutrient. Dang et al. (34) reported that pectin supplementation to piglets increased the relative abundance of *Blautia*. Conversely, Larsen et al. (35) demonstrated that pectin supplementation decreased the relative abundance of *Blautia*. As changes in the relative abundance of *Blautia* do not reflect pectin content, further analysis is required to clarify pectin utilization by *Blautia*.

### Prevalence of the clusters in human gut microbes

**Fig 6:**
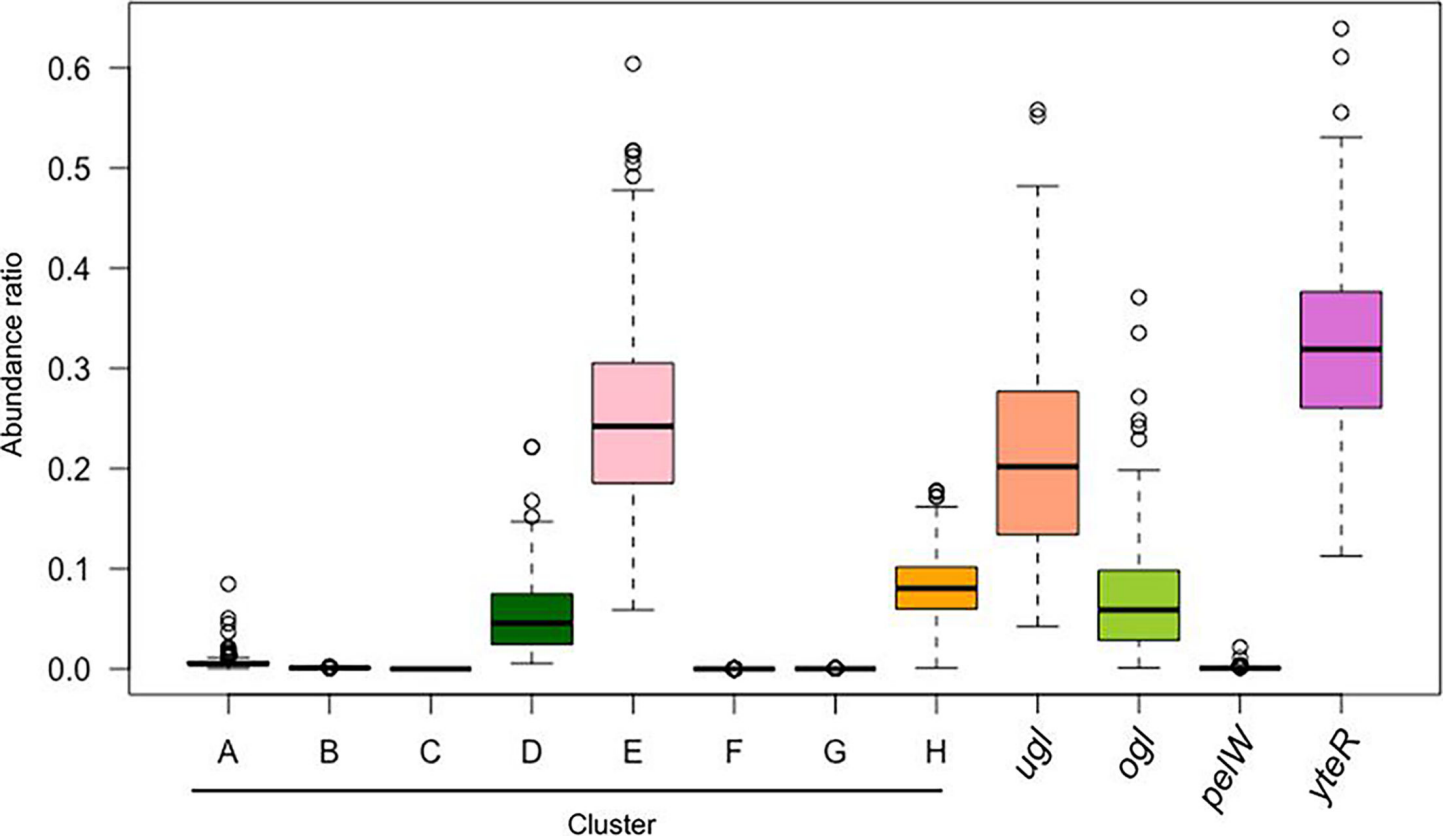
Relative abundance of the clusters in healthy adult guts. The relative abundance of types A–H and the enzyme-encoding genes (*ugl*, *ogl*, *pelW*, and *YteR*) were estimated using the metataxonomic data of 290 healthy adults in the United States.

The family Bacteroidaceae, which contains the genera Bacteroides and Parabacteroides, harbored only the type E cluster *kduI–dhuD*, which is a hybrid type of *kdu* and *dhu*. Genus *Bacteroides* is abundant in the human gut, which is rich in mucus-derived GAGs and dietary pectins. Previous reports found that *B. thetaiotaomicron* and *Bacteroides xylanisolvens* harbor specific polysaccharide utilization loci that assimilate pectin with different degrees of esterification (36–38). Therefore, we hypothesized that the presence of type E clusters in the genome is advantageous in environments where both GAGs and pectins are abundant. To examine the hypothesis, we analyzed the relative abundance of the clusters in human feces using metataxonomic data from 290 healthy adults (Fig. 6). Consistent with our hypothesis, the relative abundance of type E clusters was significantly higher than that of other types (*P* < 0.0001 by the Kruskal–Wallis and Wilcoxon tests). Regarding the phylum Bacillota, type E clusters were detected in the families Lachnospiraceae (16/34 strains), Enterococcaceae (10/23 strains), and Streptococcaceae (3/51 strains). All of these type E-positive strains, excluding the two *Lactococcus* species in the family Streptococcaceae, have been reported as human gut-commensal bacteria (39, 40), supporting our hypothesis. Moreover, UGL genes were detected at the highest rate in type E-positive strains (Fig. S1). Although OGL genes were rarely detected in type E-positive strains, they harbored YteR genes at the highest rate, in addition to UGL genes (Fig. 6). These data indicate that the possession of UGL and YteR genes is advantageous for gut colonization, similar to type E clusters.

### Habitant preference

**Fig 7:**
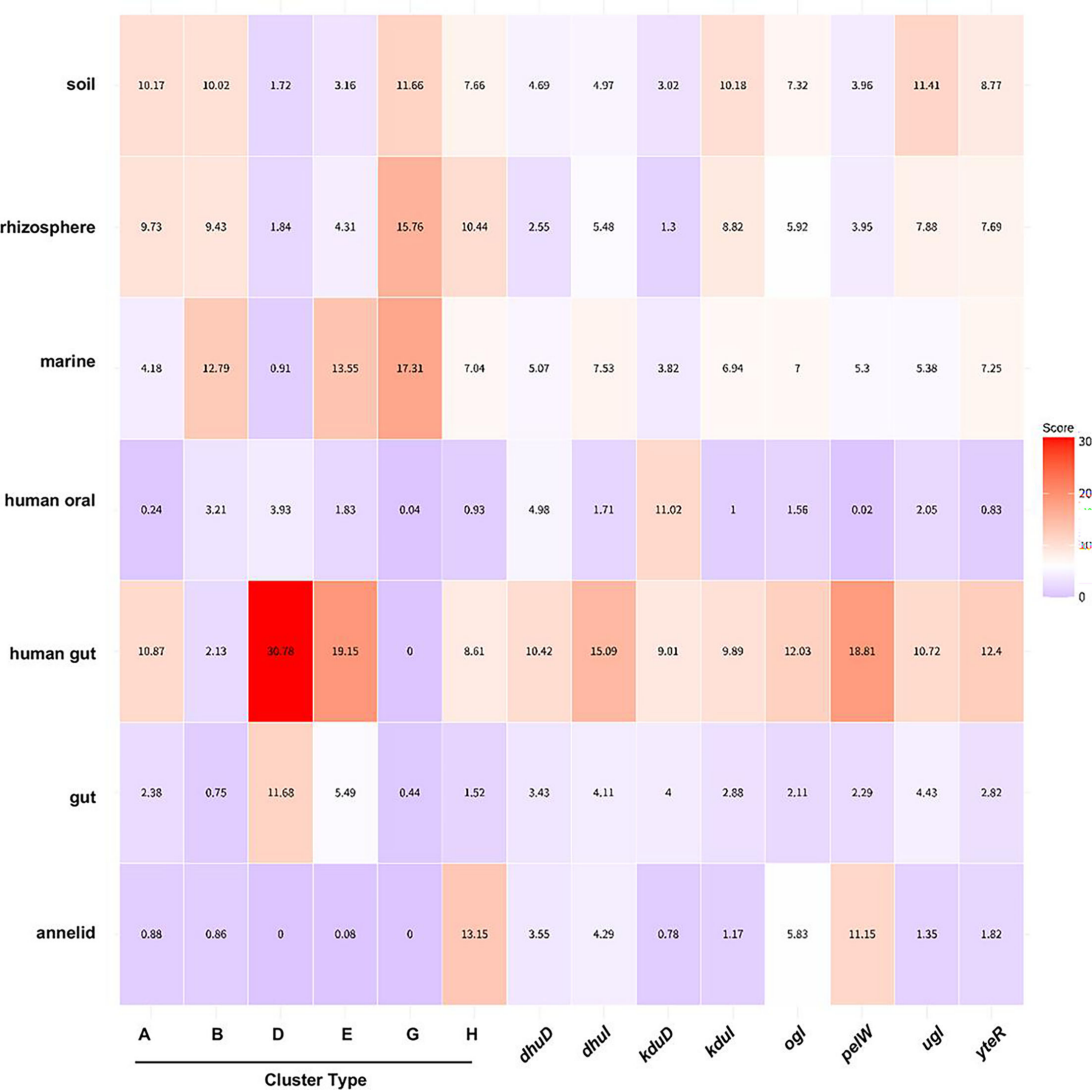
Heatmap of habitat preference scores for selected categories. Habitat preference scores were calculated using ProkAtlas (3, 41). Values inside the boxes indicate average habitant preference score. White boxes, scores of the overall average across all cluster types; red, scores above the average across all cluster types; purple, scores below the average across all cluster types. The categories (soil, rhizosphere, marine, human oral, human gut, gut, and annelid) were selected according to an average score >10 in at least one operon. Average habitat preference scores of all categories are presented in Supplementary Material (Fig. S3).

To explore whether the presence of clusters in the genome is associated with environmental adaptation, habitant preference was estimated using the ProkAtlas database (41). The average scores of habitant preference varied widely among clusters (Fig. 7; Fig. S3). Type A was commonly found in human gut, soil, and rhizosphere samples. Type D was frequently observed in the human gut but rarely detected in soil, rhizosphere, or marine environments. In contrast, type G was not detected in the human gut but tended to occur in marine, soil, and rhizosphere samples. Type E was frequently present in human gut and marine environments. Type H was broadly distributed and frequently found in the following order: annelid gut, rhizosphere, and human gut.

In the human gut, GAGs serve as major nutrient sources for the gut microbiota because they are richly present as free molecules and components of proteoglycans and they are continuously shed from the gut epithelium and mucosal layer (6). The marine environment is abundant in alginate, which is primarily produced by seaweeds (42). In soil and rhizosphere, pectin is supplied from plant cell walls, and its concentration is associated with biofilm formation and bacterial colonization (43). Our results indicated the association of clusters with environmental adaptation. Differences in substrate availability among environments might explain the distribution patterns of cluster types.

### Phylogenetic analysis based on isomerase and reductase

**Fig 8:**
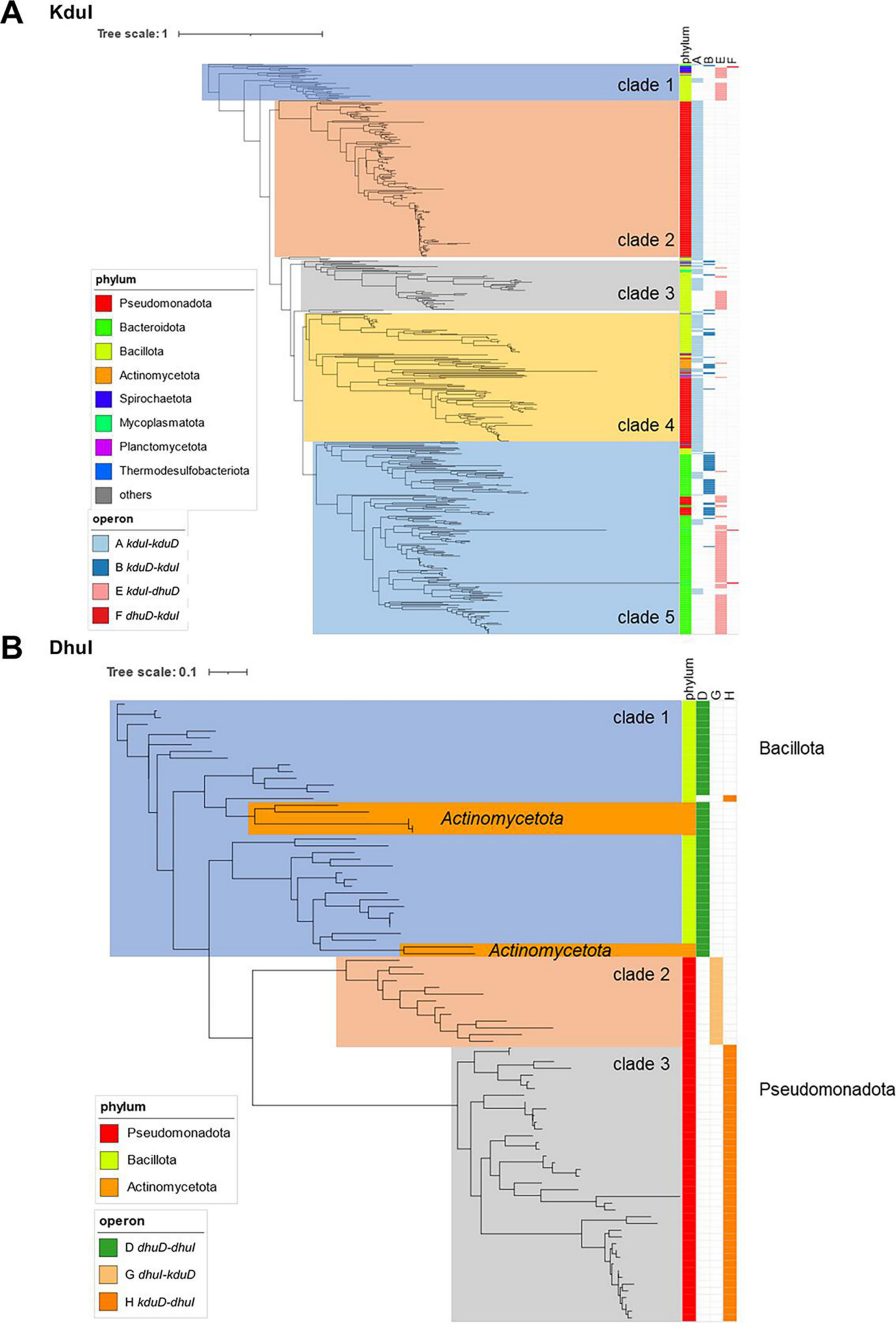
Phylogenetic tree of isomerases. (**A**) KduI. (**B**) DhuI. The tree was constructed using RaxML (42). The figure was drawn using iTOL (version 6) (30).

We conducted a phylogenetic analysis based on the amino acid sequences of isomerases and reductases (Fig. 8). Although the phylogenetic tree of KduI was constructed with five clades (Fig. 8A), the clades were not correlated with cluster types, indicating that variations in KduI sequences occurred before cluster formation. By contrast, variations in DhuI were correlated with cluster types (clade 1, type D; clade 2, type G; and clade 3, type H; Fig. 8B). This correlation indicated that the variations in DhuI occurred through different selection pressures among cluster-possessing bacteria after cluster formation. DhuI in the phylum Actinomycetota did not form an independent clade, but it clustered with that of the phylum Bacillota similarly as DhuD (Fig. S1B). The similarity of DhuI and DhuD between the different phyla suggested horizontal gene transfer of the *dhuD–dhuI* cluster from Bacillota to Actinomycetota.

### Limitations

This study had several limitations. Although we focused on DHU metabolism–related gene clusters, their presence alone does not fully explain adaptability to different environments. For instance, Browne et al. (44) reported that Firmicutes bacteria that lost sporulation capability were more abundant within individual hosts but less abundant than spore-forming Firmicutes bacteria. Similarly, Levy et al. (45) highlighted that multiple genomic traits contribute to plant-associated adaptation. As shown in Table S1, some bacteria duplicated reductase genes. In particular, *Rhodococcus pseudokoreensis* possesses 146 KduD genes, and *Mycolicibacterium moriokaense* possesses 50 DhuD genes. The large number of duplicate detections likely indicates a high rate of false positives in our gene identification approach. As homologous genes were identified using HMM and BLAST searches, it is possible that homologous proteins with different substrate specificities were incorrectly detected. As presented in Fig. 2, the isomerase and reductase genes were oriented in the same direction across types A–H clusters. In most cases, the distance between these two genes was <100 bp, except for type H (Fig. 5), suggesting that they might be conserved as clusters. However, further analysis is needed to determine whether the genes are indeed cotranscribed.

### Conclusion

This pan-genome analysis found a wide distribution of type A clusters independent of the phylum, suggesting that the type A cluster *kduI–kduD* represents the ancestral form, which was subsequently replaced during the course of evolution. The hybrid cluster *kduI–dhuD* (type E) might play a role in bacterial colonization in the intestinal environment through the metabolism of GAGs and pectins by UGLs and YteRs, respectively. Moreover, some strains harbored a tandem arrangement of three genes. This study proposes a correlation between Kdu/Dhu genes involved in the assimilation of DHU and the evolution of bacterial persistence.

## MATERIALS AND METHODS

### Data sources

Representative and complete genomes that passed the taxonomy check and their annotated protein sequences were obtained from the National Center for Biotechnology Information RefSeq database (retrieved 13 January 2023) (46) (Table S1). The genomes of 3,550 strains were collected, including two genomes derived from *Escherichia coli* (O157:H7 strain Sakai and strain K-12 substrain MG1655). Table S1 lists the accession numbers of the genomes. The genomes were classified using TaxonKit (version 0.17.0) (47). As listed in Table S3, 1,378, 653, 641, and 309 strains belonging to the phyla Pseudomonadota, Actinomycetota, Bacillota, and Bacteroidota, respectively, were included in this study.

### Phylogenetic analysis

The core genes in the 3,550 complete genomes were detected using the UBCG2 pipeline (48). Based on the concatenated sequences of the core genes, a phylogenetic tree was constructed using FastTree (49). Data sets of Bacteroidota and Bacillota were extracted to construct rooted phylogenetic trees with *Chlorobium limicola* DSM 245 and *Thermotoga maritima* strain MSB8 for the outgroups of Bacteroidota and Bacillota, respectively. Protein sequences (KduI, KduD, DhuI, and DhuD) were aligned using MAFFT (version 7.525) (50), followed by trimming of poorly aligned regions using the “automated1” method in trimAl (version 1.5.0) (51). The trimmed alignment was used as input for the phylogenetic analysis using RAxML (version 8.2.13) with the PROTGAMMALG model (52). The phylogenetic trees were visualized using iTOL (version 6) (30).

### Homologous gene search based on the HMM profile

To generate the HMM profile, 20 nonredundant sequences were selected as described by Bundalovic-Torma et al. (53) with some modifications. As initial queries, the amino acid sequences of *L. rhamnosus* strain Lc705 KduI (CAR91530.1), *L. rhamnosus* strain Lc705 KduD (CAR91531.1), *S. agalactiae* strain NEM316 DhuI (CAD47551.1), *S. agalactiae* strain NEM316 DhuD (CAD47550.1), *S. agalactiae* strain NEM316 UGL (CAD47548.1), *D. dadantii* strain 3937 OGL (ADM98639.1), *Bacillus subtilis* subsp. *subtilis* strain 168 YteR (unsaturated rhamnogalacturonyl hydrolase of the GH105 family) (CAB14990.1), *L. rhamnosus* strain Lc705 KdgA (2-dehydro-3-deoxyphosphogluconate aldolase/4-hydroxy-2-oxoglutarate aldolase) (CAR91532.1), *L. rhamnosus* strain Lc705 KdgK (2-dehydro-3-deoxygluconokinase) (CAR91533.1), and *Y. enterocolitica* subsp. *enterocolitica* strain 8081 KdgF (pectin degradation protein) (CAL11968.1) were selected (Table S2). The protein BLAST search was conducted using the annotated proteins from the 3,550 genomes as references with a threshold of *E* < 1 × 10^−5^ (28). The detected homologous sequences were collected as data sets. Among all detected homologous sequences, those with <97% sequence identity and with the lowest *E* value were selected as the next queries. The sequences of the first queries were removed from the data sets. Then, using the next queries and data sets, a protein BLAST search was conducted to detect new queries. By repeating these procedures 20 times, we obtained 21 query sequences for each protein. These selected query sequences were aligned using MAFFT (version 7.525) (50). An HMM profile was generated from the aligned sequences using HMMER (version 3.4) (54). Based on the generated profiles, homology searches were conducted using the complete genomes of 3,550 strains. Finally, genes whose complete sequences had *E* < 1 × 10^−5^ were considered homologous.

### Identification and classification of GAG metabolism–related gene clusters

To determine whether GAG isomerase and reductase are organized in a cluster, we calculated the distance between isomerase (*kduI* or *dhuI*) and reductase (*kduD* or *dhuD*) genes detected using the aforementioned HMMER search. If the distance between the gene pairs was ≤500 bp, then the pairs were defined as putative clusters. According to the types of genes and order, the putative clusters were classified into ten types (Fig. 2; Table S4 through S10).

### Fecal microbiomes of healthy adults

Metagenomic data from the fecal microbiomes of 290 healthy US adults (BioProject accession number: PRJNA795985) were analyzed in this study. Paired-end 151-bp raw sequencing reads (Run IDs, SRR17531755–SRR17532044) were retrieved using the SRA Toolkit (https://hpc.nih.gov/apps/sratoolkit.html). Quality filtering and adapter trimming were performed using fastp (55). To remove host-derived reads, sequences mapped to the human reference genome (GRCh38/hg38), which was downloaded from the UCSC Genome Browser (56), were filtered using Bowtie2 (57) and SAMtools (58). A bacterial genome database was constructed using 3,550 representative bacterial genomes, as listed in Table S1, and indexed with Kraken2 (59). Cleaned reads were classified by Kraken2, and abundance estimates were generated using Bracken (60).

### Habitat preference score estimation

rRNA gene sequences were detected using barrnap (https://github.com/tseemann/barrnap). 16S rRNA gene sequences were extracted using seqkit (61). After clustering the 16S rRNA gene sequences with a threshold of 90% sequence identity, a centroid sequence of the largest cluster was obtained using VSEARCH (62) and used as the representative 16S rRNA gene sequence. When two or more clusters contained the largest number of sequences, the longer/longest centroid sequence was used. The habitat preference score of each bacterium was calculated using ProkAtlas with default parameters based on the representative 16S rRNA gene sequences (41). Using gene cluster information listed in Table S1 (A–H, A^C^–H^C^), the average scores of habitat preference are presented in Fig. 7.

## SUPPLEMENTAL MATERIAL

Supplemental figures msphere.00817-25-s0001.docx Figures S1 to S4.

Supplemental tables msphere.00817-25-s0002.xlsx Tables S1 to S14.

ASM does not own the copyrights to Supplemental Material that may be linked to, or accessed through, an article. The authors have granted ASM a non-exclusive, world-wide license to publish the Supplemental Material files. Please contact the corresponding author directly for reuse.
