# Supplementary material for: Molecular evolution and diversity of isomerase–reductase clusters involved in the bacterial metabolism of glycosaminoglycans
Source: mSphere. 2025 Dec 29;11(1):e00817-25. doi: 10.1128/msphere.00817-25 (PMC12838403; doi:10.1128/msphere.00817-25)
Supplement: Supplemental figures — Figures S1 to S4. [file msphere.00817-25-s0001.docx]

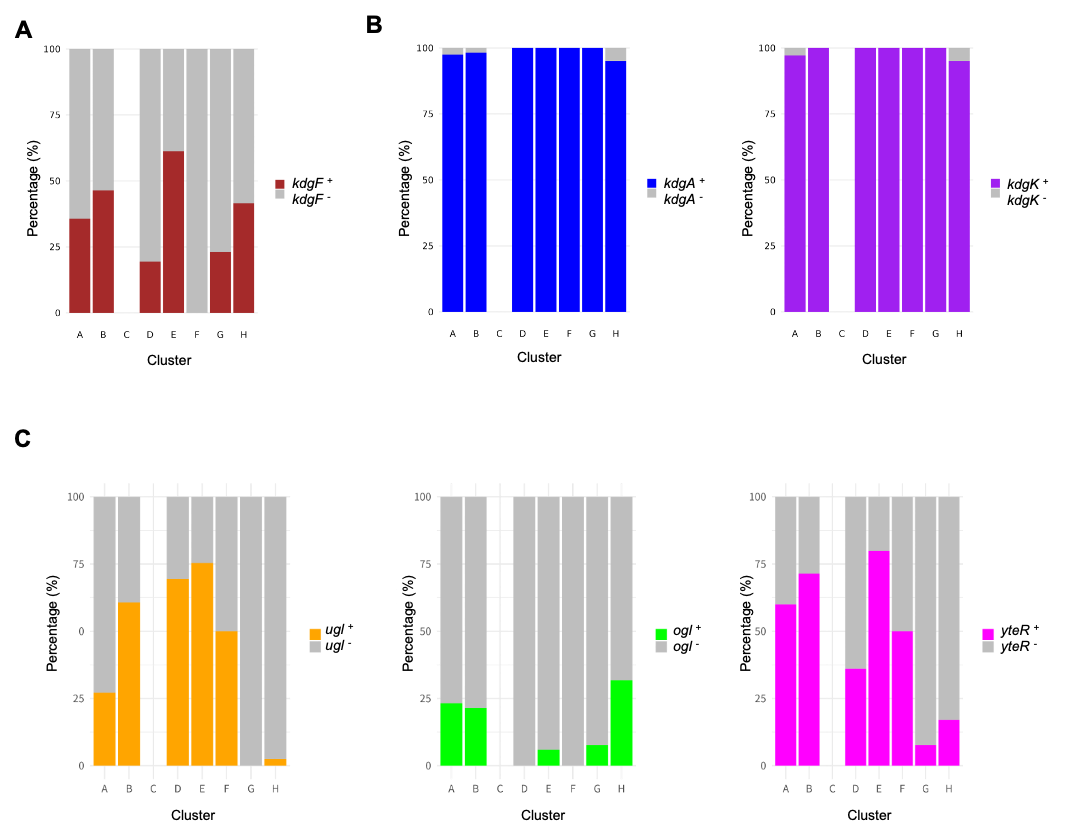


**Figure S1. Proportions of genes-positive strains among the cluster types.** (*A*) *kdgF*, (*B*) *kdgA* and *kdgK*, (*C*) *ugl* (left), *ogl* (center), and *yteR* (right) gene-positive strains


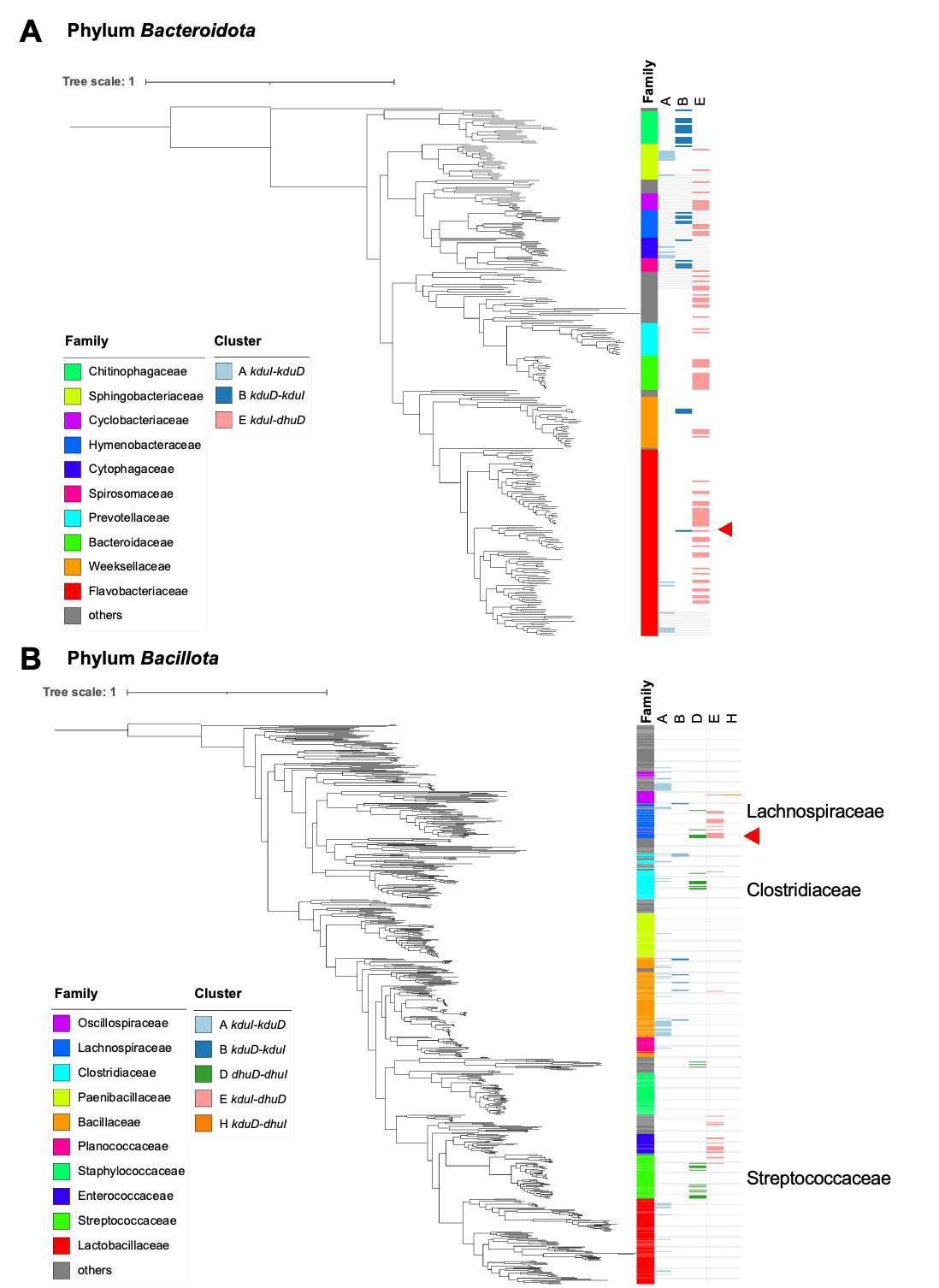


**Figure S2. Phylogenetic analysis of the two phyla.** (*A*) phylum *Bacteroidota*; (*B*) phylum *Bacillota*. The red triangles indicate clusters consisting of three genes arranged in tandem. The figure was drawn using iTOL (version 6) (51).

**
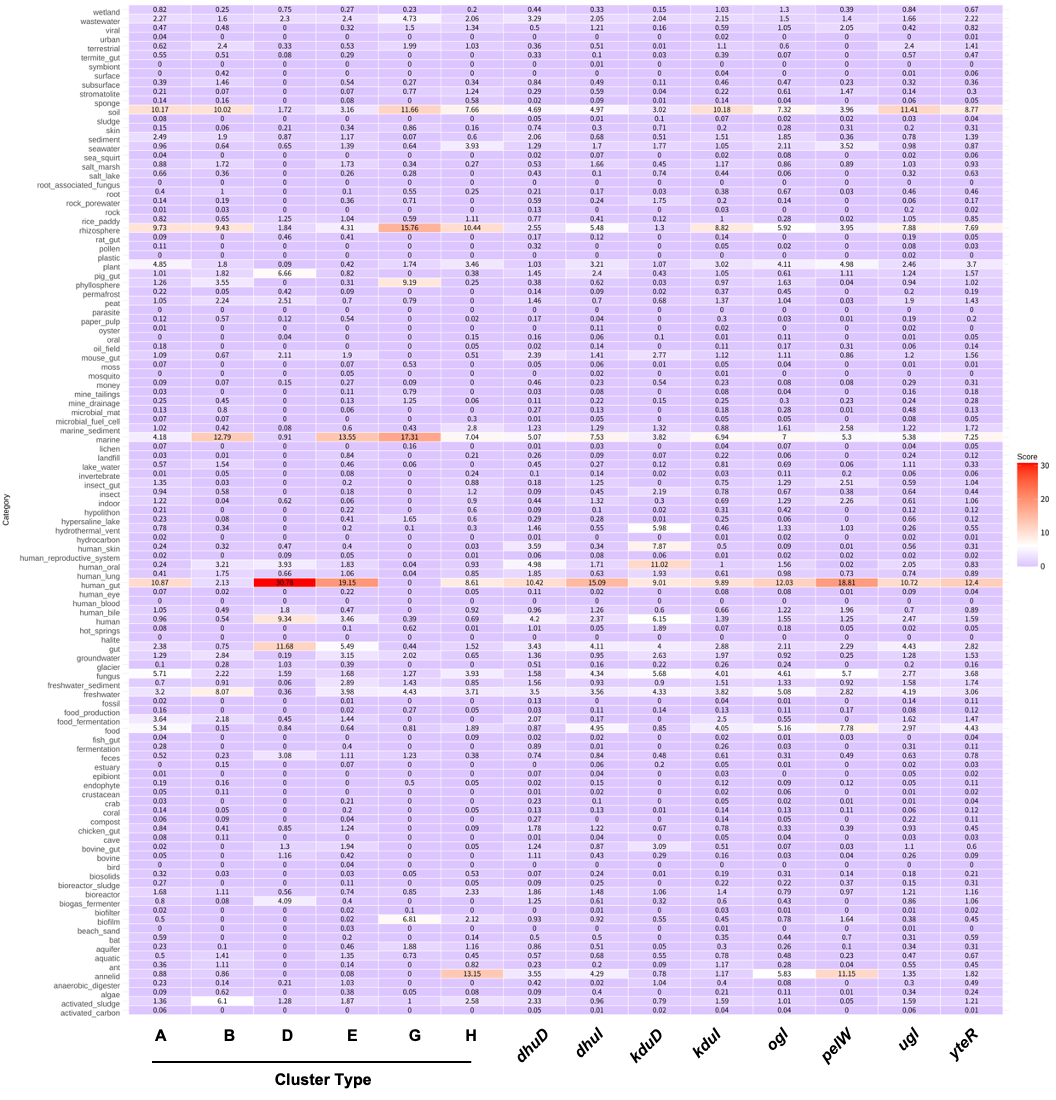
**

**Figure S3.** **Heatmap of habitat preference scores for all categories.** Habitat preference scores were calculated using ProkAtlas (31). Values inside the boxes indicate average habitant preference score. White boxes, scores of the overall average across all cluster types; red, scores above the average; purple, below the average.

**
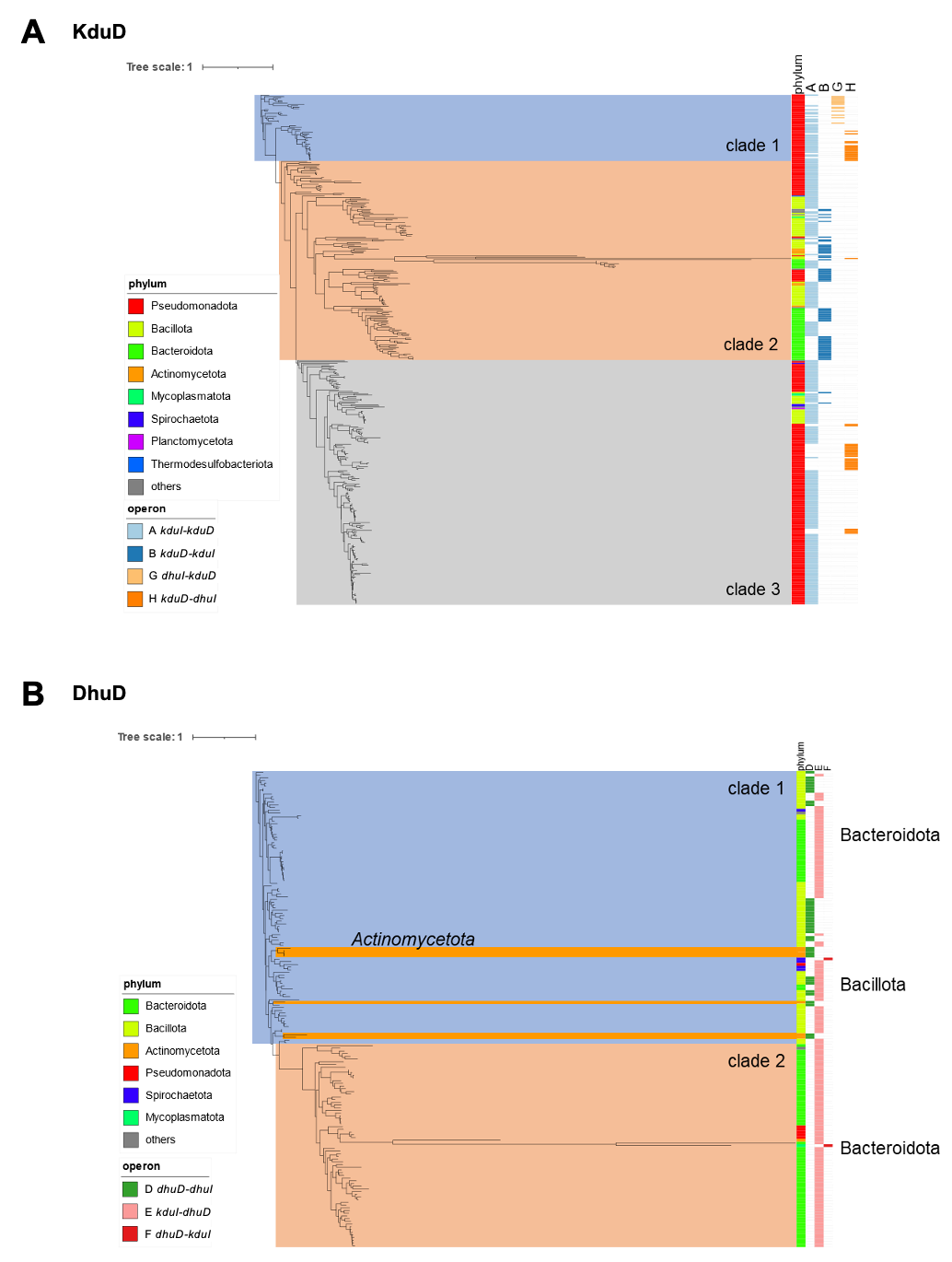
**

**Figure S4.** **Phylogenetic tree of reductases.** (*A*) KduD; (*B*) DhuD. The tree was constructed using RaxML (50). The figure was drawn using iTOL (version 6) (51).
